# Increased HSP27 correlates with malignant biological behavior of non-small cell lung cancer and predicts patient’s survival

**DOI:** 10.1038/s41598-017-13956-2

**Published:** 2017-10-23

**Authors:** Baowei Sheng, Congcong Qi, Bing Liu, Yong Lin, Tian Fu, Qingdi Zeng

**Affiliations:** Department of Respiratory Medicine, Jining NO.1 People’s Hospital, Jining, Shandong Province, China

## Abstract

Heat shock protein 27 (HSP27) has been found to be related to tumorigenesis. The aim of this study was to investigate the expression pattern and clinical significance of HSP27 in non-small-cell lung cancer (NSCLC). The expression of HSP27 in tissues was examined by immunohistochemistry and serum level of HSP27 mRNA was detected by real-time PCR. The survival analysis was performed by a Kaplan Meier method and the estimation of risk factors was determined by the multiple regression analysis. The expression of HSP27 was increased in lung cancer tissues (p < 0.001) and serum (p < 0.001) of NSCLC patients and higher HSP27 in lung cancer tissues and serum of NSCLC patients was associated with poorly differentiated cancer (p < 0.001; p = 0.035), lymphatic metastasis (p < 0.001; p < 0.001), advanced TNM stage (p < 0.001; p < 0.001). And the levels of HSP27 in tissues and serum of lung cancer patients had a certain positive correlation (p = 0.046). Moreover, increased HSP27 expression correlated with shorter survival of NSCLC patients (p < 0.001). The results suggest that HSP27 may serve as a potential biomarker for diagnosis and prognosis of NSCLC.

## Introduction

Lung cancer is the leading cause of cancer-related death in men and women in China accounting for approximately 32% of total cancer deaths in 2015 despite comprising only ~25% of new cancer cases^1^. Especially, morbidity and mortality of non-small-cell lung cancer (NSCLC) are relatively high in China, with a 5-year survival rate of less than 20%, which is often due to lung cancer first presenting at late stages and a lack of curative therapeutic options at these later stages^1^. Decades of research have contributed to our understanding that NSCLC is a multi-step process involving genetic and protein alterations which transforms normal lung epithelial cells into lung cancer^2^. It is commonly understood that an early detection of NSCLC leads to a better chance for reducing the mortality and morbidity. The advent of new and emerging molecular, genetic, and imaging technologies has broadened the possible strategies for early detection and prevention of NSCLC^3^. Molecular markers are being identified that are enhancing our ability to predict and detect NSCLC before it develops and at the earliest signs of impending carcinogenic transformation^4^. Thus, new biological markers are urgently needed to improve the early detection, diagnosis and treatment of NSCLC.

The heat shock protein 27 (HSP27), a 27 kDa protein, belongs to the heat shock protein family, which is highly expressed in some tumors and is associated with tumor metastasis, poor prognosis and resistance to chemotherapy^5^. The heat shock protein family are highly conserved molecular chaperones with indispensable roles in protein homeostasis, transport processes and signal transduction^6^. Research shows that up-regulation of HSP27 in tumor cells increases tumor cell growth, and thus most probably serves as a protective factor to chemical stress^7^. Previously, we have identified the up-regulation of HSP27 in NSCLC tissues compared with the normal lung tissues using laser capture microdissection combined with liquid chromatography-tandem mass spectrometry. However, compared with cells, investigating on the HSP27 expression in serum and tissues in NSCLC is still lacking. In this research, we tested the expression levels of HSP27 in tissues and serum of patients with NSCLC, disclosed the relationship between HSP27 and clinical parameters of NSCLC and assessed its value in discerning NSCLC.

## Results

### NSCLC tissues showed a higher expression of HSP27 than normal tissues

**Figure 1:**
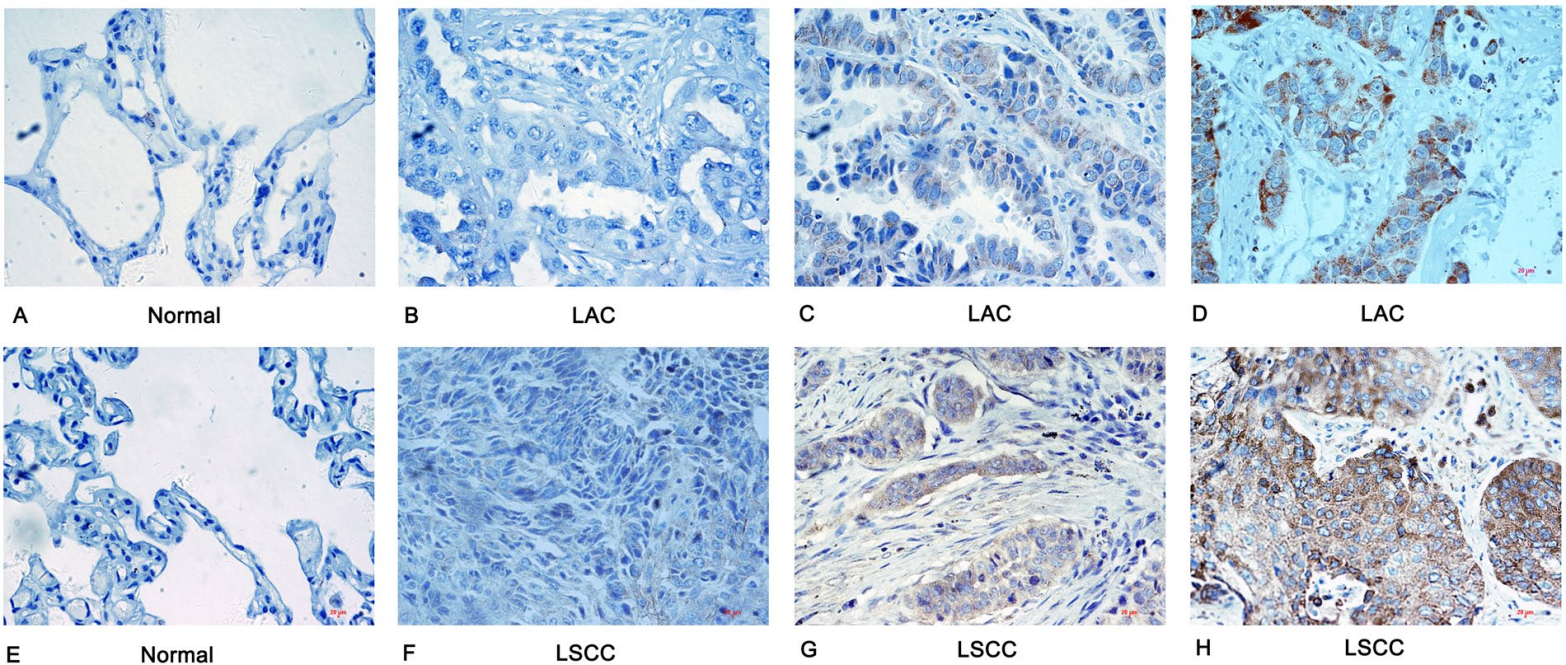
Immunohistochemistry analysis of HSP27 in NSCLC tissues and normal lung tissues (×400). (**A**) No staining of HSP27 in normal tissues. (**B**) No staining of HSP27 in well differentiated LAC. **(C)** Moderate staining of HSP27 in moderately differentiated LAC. (**D**) Intense staining of HSP27 poorly differentiated LAC. **(E)** No staining of HSP27 in normal tissues. (**F**) No staining of HSP27 in well differentiated LSCC. **(G)** Moderate staining of HSP27 in moderately differentiated LSCC; (**H**) Intense staining of HSP27 in poorly-differentiated LSCC; NSCLC, non-small cell lung cancer; LAC, lung adenocarcinoma; LSCC, lung squamous cell carcinoma.

**Figure 2:**
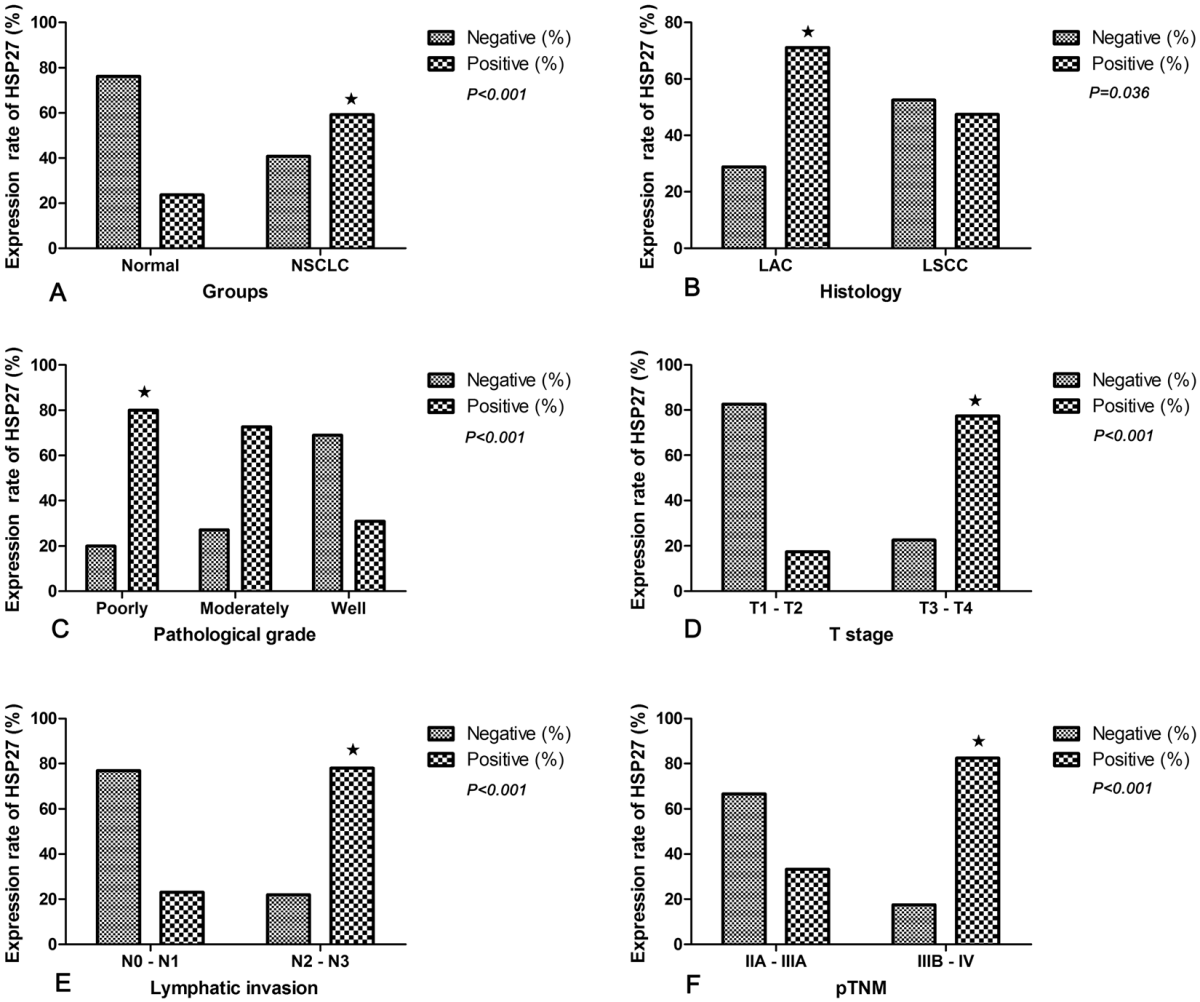
Correlation between the expression of HSP27 in tissues and clinicopathologic factors of NSCLC patients. (**A**) Higher positive expression of HSP27 in NSCLC tissues than in normal tissues. (**B**) Higher positive expression of HSP27 in LAC tissues than in LSCC tissues. (**C**) Higher positive expression of HSP27 in poorly differentiated NSCLC tissues than in moderately and well tissues differentiated NSCLC tissues. (**D**) Higher positive expression of HSP27 in NSCLC tissues at T3-T4 stage than in cancer tissues at T1-T2 stage. (**E**) NSCLC tissues with lymph node metastasis (N2-N3) revealed higher positive expression of HSP27 than lymph node metastasis (N0-N1). (**F**) NSCLC tissues at staged IIIB-IV had higher positive expression of HSP27 than those at stages IIA- IIIA; NSCLC, non-small cell lung cancer; LAC, adenocarcinoma of the lung; LSCC, squamous cell carcinoma of the lung; T, the size of the tumor mass; N, node stage (TNM classification).

The immunohistochemistry (IHC) method was employed to detect the expression of HSP27 in lung cancer tissues and cancer adjacent normal tissues of 76 NSCLC patients. We found that HSP27 was expressed in the cytoplasm of lung cancer cells and normal cells. Cells that appeared to have brown-yellow granules in the nuclei or cytoplasm were considered to be positive expression (Fig. 1A–H). The statistical results showed that lung cancer tissues showed a higher expression of HSP27 (45/76, 59.2%) than the cancer adjacent normal tissues (18/76, 23.7%) (P < 0.001) (Table 1 and Fig. 2A).

**Table 1:** Correlation between clinico-pathological features and the expressions of HSP27 in NSCLC. LAC, lung adenocarcinoma; LSCC, lung squamous cell carcinoma; Smoking, pack years of smoking; T stage, tumor size; pTNM, clinical stage of lung cancer.

**Parameter**	**Group**	**N**	**Expressions of HSP27 in lung tissues**			
			**Negative (%)**	**Positive (%)**	**χ** ^**2**^ **value**	***P*** **value**
Gender						
	Normal	76	58(76.3)	18(23.7)	19.76	<0.001
	NSCLC	76	31(40.8)	45(59.2)		
Gender						
	Male	60	26(43.3)	34(56.7)	0.764	0.382
	Female	16	5(31.3)	11(68.8)		
Ages						
	<60	34	15(44.1)	19(55.9)	0.282	0.595
	≥60	42	16(38.1)	26(61.9)		
Smoking						
	0	45	15(33.3)	30(66.7)	3.238	0.198
	0.1–40	6	4(66.7)	2(33.3)		
	>40	25	12(48)	13(52)		
Histology						
	LAC	38	11(28.9)	27(71.1)	4.413	0.036
	LSCC	38	20(52.6)	18(47.4)		
Pathological grade						
	Poorly	25	5(20)	20(80)	15.671	<0.001
	Moderately	22	6(27.3)	16(72.7)		
	Well	29	20(69)	9(31)		
T stage						
	T1–T2	23	19(82.6)	4(17.4)	23.882	<0.001
	T3–T4	53	12(22.6)	41(77.4)		
Lymphatic invasion						
	N0–N1	26	20(76.9)	6(23.1)	21.364	<0.001
	N2–N3	50	11(22)	39(78)		
pTNM						
	IIA–IIIA	36	24(66.7)	12(33.3)	18.965	<0.001
	IIIB–IV	40	7(17.5)	33(82.5)

### Increased HSP27 in NSCLC tissues correlated with clinicopathologic factors of NSCLC

As shown in Table 1, HSP27 was highly expressed in lung adenocarcinoma (LAC) (27/38, 71.1%), compared with lung squamous cell carcinoma (LSCC) (18/38, 47.4%) (P = 0.036). Also, poorly differentiated NSCLC tissues (20/25, 80%) showed an elevated HSP27 expression compared with the well differentiated tissues (9/29, 31%) (*P* < 0.001). In addition, overexpression of HSP27 was observed in NSCLC tissues with T3-T4 (41/53, 77.4%), but not in those of T1-T2 (4/23, 17.4%) (P < 0.001). Moreover, NSCLC tissues with lymph node metastasis (N2-N3) (39/50, 78%) displayed a higher expression of HSP27 than those with lymph node metastasis (N0-N1) (6/26, 23.1%) (P < 0.001). Furthermore, an increase of HSP27 was exhibited in tissues with NSCLC at stage IIIB-IV (33/40, 82.5%), compared with those of stage IIA-IIIA (12/36, 33.3%) (P < 0.001) (Table 1 and Fig. 2B–F). However, the expressions of HSP27 was irrelevant to gender, age and smoking of NSCLC patients.

**Table 2:** Correlation between clinico-pathological features and the expressions of HSP27 mRNA in serum of NSCLC. M ± SD, mean±standard deviation; LAC, lung adenocarcinoma; LSCC, lung squamous cell carcinoma; Smoking, pack years of smoking; ^★^poor and moderate differentiation compared with well differentiation; T stage, tumor size; pTNM, clinical stage of lung cancer.

**Parameter**	**Group**	**N**	**Expressions of HSP27 mRNA in serum of NSCLC**			
			**2** ^−ΔΔCt^ **(M ± SD)**	**Degree of freedom**	**Statistical value**	***P*** **value**
Gender						
	Normal	76	42.28± 14.28	112	7.063	<0.001
	NSCLC	76	23.66± 10.93			
Gender						
	Male	60	42.71± 15.19	74	0.502	0.617
	Female	16	40.68± 10.46			
Ages						
	<60	34	41.26 ± 11.89	74	0.557	0.579
	≥60	42	43.10 ± 16.06			
Smoking						
	0	45	41.66± 13.49	73	0.127	0.881
	0.1–40	6	44.36± 21.78			
	>40	25	42.90± 14.28			
Histology						
	LAC	38	43.60± 12.62	74	0.807	0.422
	LSCC	38	40.95± 15.84			
Pathological grade						
	Poorly	25	45.61± 11.30 ^★^	73	3.498	0.035
	Moderately	22	45.54± 14.04 ^★^			
	Well	29	36.94± 15.55			
T stage						
	T1–T2	23	31.76± 9.93	74	4.814	<0.001
	T3–T4	53	46.84± 13.5			
Lymphatic invasion						
	N0–N1	26	32.51± 8.96	74	4.917	<0.001
	N2–N3	50	47.36± 13.94			
pTNM						
	IIA–IIIA	36	34.81± 9.72	74	4.955	<0.001
	IIIB–IV	40	48.99 ± 14.49			

### NSCLC patients presented a higher level of HSP27 mRNA in serum than benign lung disease patients

**Figure 3:**
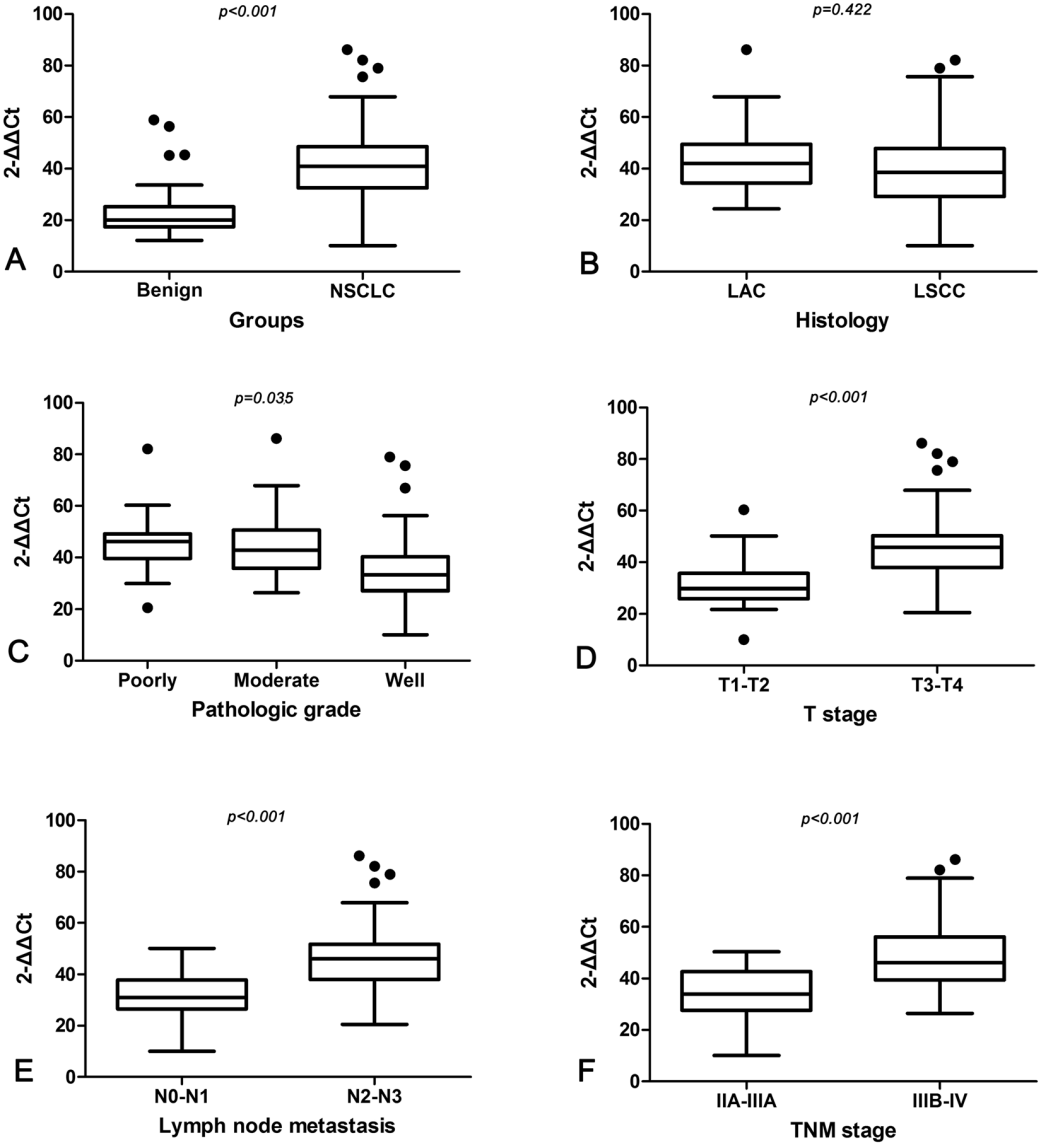
Correlation between the serum level of HSP27 mRNA and clinicopathologic factors of lung cancer patients. (**A**) Increased serum level of HSP27 mRNA in NSCLC patients than in benign lung disease patients. (**B**) The level of serum HSP27 mRNA did not have a difference between LAC and LSCC patients. (**C**) Higher level of serum HSP27 mRNA in poorly differentiated NSCLC than in moderately and well tissues differentiated NSCLC. (**D**) Higher level of serum HSP27 mRNA in NSCLC at T3-T4 stage than at T1-T2 stage. (**E**) NSCLC patients with lymph node metastasis (N2-N3) revealed a higher level of serum HSP27 mRNA than patients with lymph node metastasis (N0-N1). (**F**) NSCLC patients at staged IIIB-IV had higher level of serum HSP27 mRNA than those at stages IIA-IIIA; NSCLC, non-small cell lung cancer; LAC, adenocarcinoma of the lung; LSCC, squamous cell carcinoma of the lung; T, the size of the tumor mass; N, node stage (TNM classification).

Serum level of HSP27 mRNA was detected by a method of real-time PCR in a series of 76 NSCLC patients and a series of 38 benign lung disease patients. The results showed that expression level of serum HSP27 mRNA was higher in patients with NSCLC (42.28 ± 14.28, 2^−ΔΔCt^) than that in benign lung disease patients (23.66 ± 10.93, 2^−ΔΔCt^) (P < 0.001) (Table 2, Fig. 3A).

### Increased serum HSP27 mRNA correlated with clinicopathologic factors of NSCLC patients

As shown in Table 2 and Fig. 3B–F, patients with poorly differentiated NSCLC showed an elevated serum level of HSP27 mRNA (45.61 ± 11.30, 2^−ΔΔCt^) compared with the patients with well differentiated NSCLC (36.94 ± 15.55, 2^−ΔΔCt^) (P = 0.035). In addition, patients with T3-T4 (46.84 ± 13.5, 2^−ΔΔCt^) revealed a higher serum level of HSP27 mRNA than those at T1-T2 stage (31.76 ± 9.93, 2^−ΔΔCt^) (P < 0.001). And patients with N2-N3 of lymph node metastasis (47.36 ± 13.94, 2^−ΔΔCt^) exhibited an increased serum level of HSP27 mRNA than those with N0-N1 (32.51 ± 8.96, 2^−ΔΔCt^) (P < 0.001). Furthermore, NSCLC patients at IIIB-IV stage (48.99 ± 14.49, 2^−ΔΔCt^) displayed a higher serum level of HSP27 mRNA than those at IIA-IIIA stage (34.81 ± 9.72, 2^−ΔΔCt^) (*P* < 0.001). But the serum level of HSP27 mRNA did not correlate with gender, age, smoking and histological classification of NSCLC patients.

### Expression level of HSP27 protein in tissues was consistent with the expression level of HSP27 mRNA in serum

**Figure 4:**
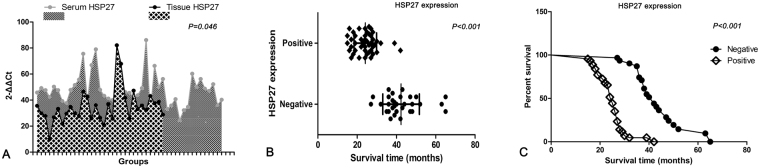
Consistency test of HSP27 expression and Kaplan-Meier survival analysis. (**A**) HSP27 expression in tissues was consistent with the level of HSP27 mRNA in serum (McNemar test, P = 0.046). (**B**) Scatter diagram showed that higher expression of HSP27 correlated with early emergence of death in NSCLC; **(C)** Survival curve showed that positive expression of HSP27 was associated with a shorter post-surgery survival time (P < 0.001).

To verify whether the HSP27 expression in lung cancer tissues is consistent with serum HSP27 mRNA of patients, we performed a non-parametric rank correlation analysis in matched 76 tissues and serum specimens. We divided the serum detection value of HSP27 mRNA into two zones (low and high), which corresponded to the two grades of tissues detection. The statistical analysis showed that the correlation coefficient (*spearman* rank test) of expression level of HSP27 protein in tissues and the expression level of HSP27 mRNA in serum is 0.697 (*P* = 0.046), suggesting that tissues HSP27 and serum mRNA in patients with NSCLC had a certain positive correlation (Fig. 4A).

### Higher level of HSP27 expression was related to the shorter survival of NSCLC patients

Through medical follow-up, we got the survival data from the patients. The definition of survival time is as follows: start time = the date of taking tissues samples (the time of surgery); terminating time = the date of patients’ death (or the last date lost to follow-up). A total of 69 out of 76 patients had follow-up records, which were included in survival analysis. The Kaplan-Meier curves analysis showed that patients who displayed a high expression of HSP27 appeared to have a shorter survival after surgery, as compared with patients with low level of HSP27 (P < 0.001) (Fig. 4B and C). In addition, lymph node metastasis (P = 0.037) and advanced TNM stage (P < 0.001) were also shown to be important risk factors which reduced the survival time of NSCLC patients. Cox-Regression analysis provided the final risk function: H(t) = [h_0_(t)]e^(0.922 ×6+1.99 ×8+1.657 ×9)^ (Table 3).

**Table 3:** Cox proportional hazards regression model analysis of overall survival. LAC, lung adenocarcinoma; LSCC, lung squamous cell carcinoma; Smoking, pack years of smoking; TNM, clinical stage of lung cancer; OR, odds ratio; CI, confidence interval.

**Variables (X)**	**Categories (different groups)**	**P value**	**OR value**	**95% CI for OR**	
				**lower**	**upper**
Gender (X1)	Male (X_1−0_) vs. female (X_1−1_)	0.700	—	—	—
Age (X2)	<60 (X_2−0_) vs. ≥60 (X_2−1_)	0.422	—	—	—
Smoking (X3)	0 (X_3−0_) vs. 0.1–40 (X_3−1_) vs. > 40 (X_3−2_)	0.713	—	—	—
Histology (X4)	LAC (X_4−0_) vs. LSCC (X_4−1_)	0.256	—	—	—
Differentiation (X5)	Poor (X_5−0_) vs. moderate (X_5−1_) vs. well (X_5−2_)	0.106			
Lymphatic invasion (X6)	N0-N1(X_6−0_) vs. N2-N3 (X_6−1_)	0.037	1.34	1.017	1.762
T stage (X7)	T1-T2 (X_7−0_) vs. T3-T4 (X_7−1_)	0.321			
TNM (X8)	IIA–IIIA (X_8−0_) vs. IIIB–IV (X_8−1_)	<0.001	5.24	2.586	10.622
HSP27 (X9)	Negative (X_9−0_) vs. Positive (X_9−1_)	<0.001	2.69	1.163	6.232
Risk function:		H(t) = [h_0_(t)]e^(0.922 ×6+1.99 ×8+1.657 ×9)^			

### Threshold value of serum HSP27 mRNA and accuracy analysis for discerning patients with NSCLC

**Figure 5:**
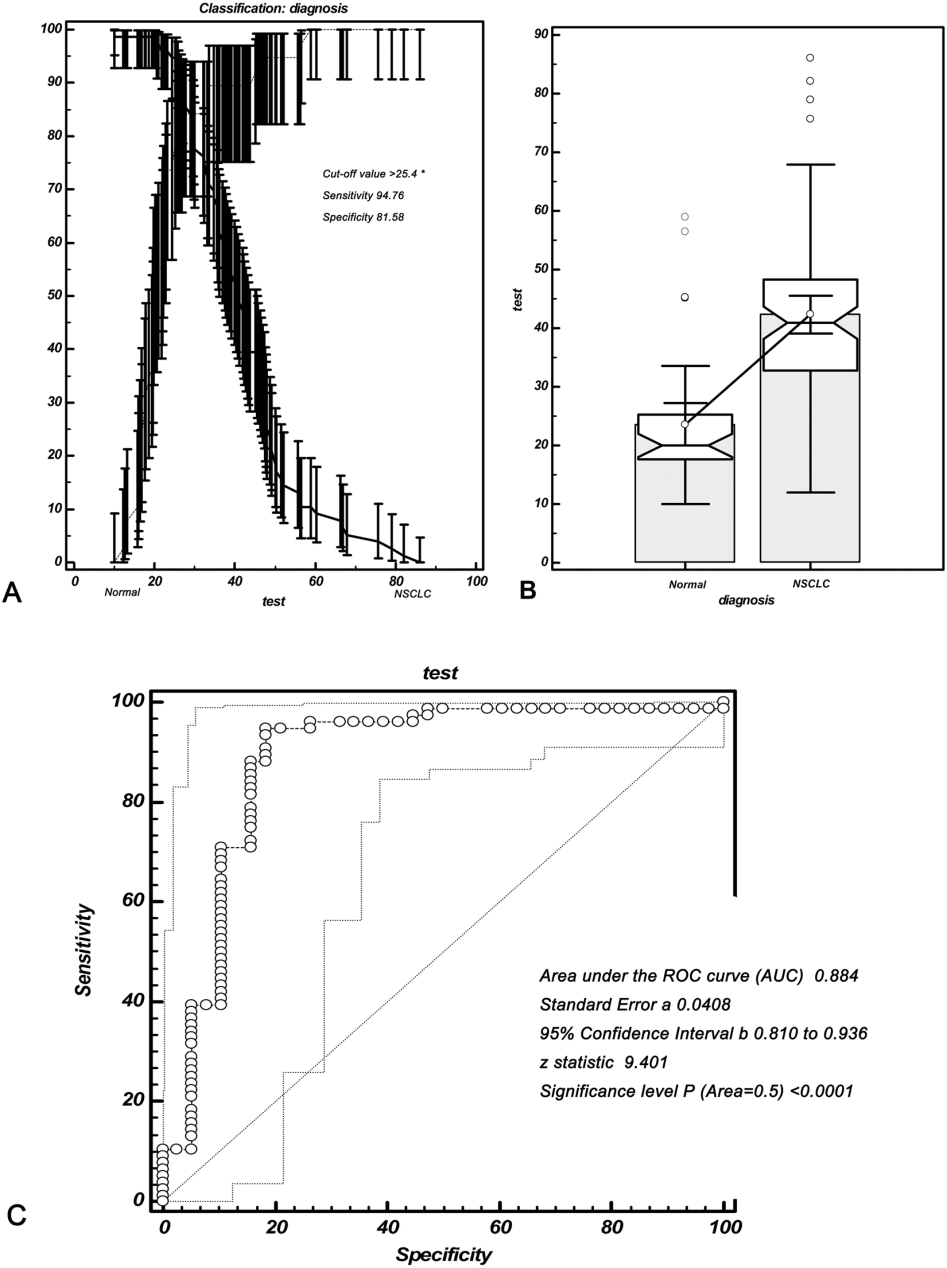
Diagnostic accuracy of serum HSP27 mRNA for NSCLC. (**A**) After calculating, the cut-off value (25.4, 2^−ΔΔCt^) of serum HSP27 mRNA appeared to discern the NSCLC patients from benign lung disease, responding a sensitivity of 94.76% and specificity of 81.58%. (**B**) The expression of serum HSP27 mRNA in NSCLC group was significantly higher than that in normal group; the minimum value of HSP27 mRNA was 10 (2^−ΔΔCt^), which was observed in the benign lung disease group while the maximum was 82.11 (2^−ΔΔCt^), located in the disease group with NSCLC; there were 3 samples in the normal group and 4 samples in the NSCLC group are out of the dispersion. (**C**) Receiver operating characteristic curves (ROC) of serum HSP27 mRNA for distinguishing NSCLC patients from benign lung disease (AUC = 0.884); ROC, receiver operating characteristic curve; AUC, area under the curve.

The threshold value of serum HSP27 mRNA for discerning patients with NSCLC from benign lung diseases was determined via ROC curve analysis. The minimum value of HSP27 mRNA was 10 (2^−ΔΔCt^), which was observed in the benign lung disease group while the maximum was 82.11 (2^−ΔΔCt^), located in the disease group with NSCLC (Fig. 5A and B). The threshold of serum HSP27 mRNA for distinguishing NSCLC patients from benign lung diseases was defined as 25.4 (2^−ΔΔCt^), responding a sensitivity of 94.76% and specificity of 81.58% (Table 4). In order to discern NSCLC, the AUC (area under the curve) of serum HSP27 mRNA arrived at 0.884 with 0.0408 of standard error, while 95% CI was from 0.810 to 0.936, and then Z value was 9.401 (*P* < 0.001) (Fig. 5C).

**Table 4:** Cut-off score of serum HSP27 mRNA level for differentiating lung cancer patients from benign lung diseases. 95% CI, 95% confidence.

**Cut-off value of serum HSP27 mRNA for discerning lung cancer patients from benign lung disease patients**						
**Criterion**	**Sensitivity**	**95% CI**	**Specificity**	**95% CI**	** + LR**	**−LR**
> = 10	100.00	95.3–100.0	0.00	0.0–9.3	1.00	
>16.4	98.68	92.9–100.0	18.42	7.7–34.3	1.21	0.071
>19.8	98.68	92.9–100.0	50.00	33.4–66.6	1.97	0.026
>21.89	96.05	88.9–99.2	57.89	40.8–73.7	2.28	0.068
>22.8	96.05	88.9–99.2	68.42	51.3–82.5	3.04	0.058
>23.1	96.05	88.9–99.2	73.68	56.9–86.6	3.65	0.054
>24.37	94.74	87.1–98.5	73.68	56.9–86.6	3.60	0.071
>25.4 *	94.76	87.1–98.5	81.58	65.7–92.3	5.14	0.065
>26.45	89.47	80.3–95.3	81.58	65.7–92.3	4.86	0.13
>28.99	84.21	74.0–91.6	84.21	68.7–94.0	5.33	0.19
>32.36	75.00	63.7–84.2	84.21	68.7–94.0	4.75	0.30
>36.13	61.84	50.0–72.8	89.47	75.2–97.1	5.88	0.43
>45.55	38.16	27.2–50.0	94.74	82.3–99.4	7.25	0.65
>49.2	21.05	12.5–31.9	94.74	82.3–99.4	4.00	0.83
>58.9	10.53	4.7–19.7	100.00	90.7–100.0		0.89

## Discussion

Lung cancer is the leading cause of cancer death worldwide. Approximately 80% of lung cancers are grouped as NSCLC, which are clinically and pathologically different from small cell lung carcinoma (SCLC)^8^. Recent advances in lung cancer research have enabled significant progress in our understanding of the molecular pathogenesis and treatment for lung cancer^9^. These discoveries graphically illustrate that molecular biological findings are directly linked to the development of clinical oncology and contribute to improve the survival rates of patients with lung cancer^10^. Heat shock protein 27 (HSP27) also known as heat shock protein beta-1 (HSPB1), which is encoded by the HSPB1 gene. HSP27 is an ATP-independent molecular chaperone, is induced by the heat shock associated with physical and chemical stresses, including radiation, oxidative stress, and various chemotherapies. HSP27 has been appeared to be constitutively expressed at high levels in various tumors, including gastric, prostate, and pancreatic cancer^11^. Study shows that elevated expression of HSP27 in cancers has been associated with poor clinical prognosis and therapeutic resistance and has been suggested as a diagnostic, predictive or prognostic marker in various tumors^12^. In the current study, we identified the expressions of HSP27 in NSCLC tissues and serum of patients, and analyzed the clinical significance of it expression and the potential use of HSP27 as clinical markers for the diagnosis and prognosis of NSCLC.

We found that whether in lung cancer tissues or in serum of patients, the expression of HSP27 was up-regulated in NSCLC, particularly in poorly differentiated lung cancer. In clinical practice, poorly differentiated lung cancers tend to progress faster and have a poor prognosis than moderately and well differentiated tumors^4^. Thus, we can infer that high expression of HSP27 is involved in tumorigenesis of NSCLC and potentially promotes NSCLC development. Previous research suggest that over-expression of HSP27 is associated with promoting drug resistance, aggressive cancers, metastasis, and poor patient outcomes and influence cellular processes such as apoptosis, DNA repair, recombination, and formation of metastases^13^. In the study of NSCLC tissue level, we found that HSP27 expression was significantly higher in lung adenocarcinoma than in squamous cell carcinoma. But, this phenomenon was not verified in the subsequent serological studies. However, this conclusion still has a specific clinical indication that the high expression of HSP27 in NSCLC may be more relevant to lung adenocarcinoma. Some previous studies have shown that HSP27 expression was significantly increased in some different tumors of adenocarcinoma^14–16^. In addition, we found that NSCLC tissues with the lymph node metastasis showed a high expression of HSP27 and the up-regulation of HSP27 intimately correlated with the tumor size positively, which means that overexpression of HSP27 was likely to facilitate the lymphatic metastasis of lung cancer and disease progression. Our studying conclusion is consistent with previous some studies, in which overexpression of HSP27 usually leads to a poor prognosis of patients and is intimately related to the malignant biological characteristics of cancers^5–7,12–15^. Previous studies also suggest that there is a significant correlation between serum Hsp70 levels and the gross tumor volume of NSCLC^17^ and serum levels of HSP27 are elevated in patients with NSCLC diagnosed at an early or advanced stage when compared with healthy control groups^18^. These conclusions suggest that both in lung cancer and in patients with serum, HSP27 expression levels are upregulated, indicating that HSP27 is closely related to the occurrence and development of lung cancer. Invasion and metastasis are the main characteristics during the progression of malignant tumors, which is responsible for the majority of cancer mortalities. Lymph node metastasis is an important prognostic indicator in a number of cancer types. More than half of NSCLC patients have lymph node metastasis when they are initially diagnosed or underwent surgical resection, which results in poor prognosis^19,20^. Similarly, research reports that tumor size is an independent prognostic factor, for early stage as well as node positive and locally invasive disease, and tumor size had significantly better predictive accuracy than alternative model without tumor size^21^. Our subsequent serological study also confirmed that increased HSP27 mRNA in serum of NSCLC patients were significantly associated with lymph node metastasis and advanced T stage. The another discovery from our study is also in accordance with previously published investigations, where an up-regulated level of HSP27 in malignant tumors is associated with advanced TNM classification, suggesting that increased expression of HSP27 contributes to the progression and deterioration of NSCLC^5,12,16^. And it is a clear conclusion that advanced TNM classification predicts a poor outcome and a shorter survival of malignant tumors^21^. Previous studies find that HSP27 plays an important role in the development of NSCLC through complex upstream and downstream signal control. The tyrosine kinase encoded by the MET oncogene is activated by gene mutation or amplification in tumors. MET kinase inhibition resulted in a 3- to 4-fold increased expression of HSP27. HSP27 increase depended on the inhibition of the MEK/ERK pathway and on heat-shock factor 1 (HSF1) and hypoxia-inducible factor-1α (HIF-1α) regulation^22^. Transforming growth factor β (TGF-β) signal pathway abnormality is widely observed in drug resistance during lung cancer chemotherapy. TGF-β induces the mRNA and protein expression of HSP27 in human lung cancer cell (A549) and the knockdown of HSP27 blocks TGF-β-induced cisplatin resistance via decreasing cell viability and increasing cell apoptosis in A549 cell^23^. It is reported that HSP27 is highly expressed in lung cancer tissues from mice, as well as in a lung cancer cell line. In the *in vitro* model, the overexpression of HSP27 promotes cell proliferation by upregulating specific target genes, which requires the activation of the activator protein-1 (AP-1) signaling pathway following the phosphorylation of c-Jun^24^. Epithelial to mesenchymal transition (EMT) is induced by transforming growth factor-β1 (TGF-β1) and is a crucial event for cancer cells to acquire invasive and metastatic phenotypes. The silencing of HSP27 enhances an additive effect with TGF-β1-induced EMT and the TGF-β1-induced HSP27 increase is not affected by the suppression of Smad2 and Smad3 in A549 cells, which suggests that HSP27 is involved in TGF-β1-induced EMT in a Smad-independent manner in lung cancer cells^25^.

A previous meta-analysis suggests that HSP27 expression is significantly associated with the unfavorable conditions for differentiation degree, lymphatic metastasis, clinical stage and tumor size. Meanwhile, HSP27 expression seems to be a predictor for a lower 5-year overall survival (OS) rate but not for 1-year OS of NSCLC^26^. Our findings are consistent with the conclusion of the meta-analysis. To analyze the prognostic impact of HSP27 overexpression on the survival of NSCLC patients, we also performed a multifactor retrospective analysis. We found that the overexpression of HSP27 appeared to be an important independent risk factor correlated with poor prognosis of NSCLC patients, which suggests that the NSCLC patients with higher expression of HSP27 (24.3± 5.7 months) have a shorter survival time compared with patients with the low expression of HSP27 (42.4 ± 9.3 months) (risk coefficient = 1.657). In addition to HSP27, the other two variables were also introduced into the regression equation: lymph node metastasis and advanced TNM stage. The final risk function was H(t) = [h_0_(t)]e^(0.922 ×6+1.99 ×8+1.657 ×9)^, suggesting that these factors all affect the outcome of NSCLC patients and their risk coefficients were reached at 0.922 and 1.99 respectively. As we all know, once lymph node metastasis and distant metastasis take place, the survival time of patient with malignant tumors will be remarkably shortened, and will also lead to a great adverse to the treatment^27^. Through our results, it is suggested that HSP27 can be used as a new tumor marker to evaluate the prognosis of NSCLC. Many studies have shown that HSP27 overexpression correlates with cell proliferation, motility and metastasis of malignant tumors; however, anti-HSP27 treatment has shown to be effective in suppressing the growth and migration of cancer cells^6,12,13,28^. These results indicate that HSP27 may serve as a valid prognostic marker and therapeutic target for NSCLC. And the characterization of HSP27 expression in NSCLC will conduce to clarify a new molecular pathway on the tumourigenesis and progression of NSCLC and may lead to the development of molecular diagnostic tool and target-specific therapy for NSCLC.

Because this study also involved serological test of HSP27, we analyzed whether serum HSP27 mRNA can discern NSCLC patients from normal individuals. The results showed that the serum HSP27 mRNA had an ideal ability for discerning NSCLC with a sensitivity of 94.76% and specificity of 81.58%, indicating that it can be a useful biomarker for NSCLC diagnosis. We calculated the threshold value of serum HSP27 mRNA through drawing the ROC curve, which showed HSP27 mRNA is conduce to discern the NSCLC. Using an univariate logistic regression models including healthy subjects and patients with NSCLC, previous study finds that HSP27 shows an AUC of 0.870 (P < 0.0001) and suggests that serum HSP27 levels might serve as a possible tool to discriminate between early and advanced stages NSCLC^18^. So far, a number of studies are concordant with the notion that HSP27 expression is up-regulated in a variety of human cancers, and may be served as effective prognostic and diagnostic indicators for cancer patients^5–7,12–15^. However, multi-center and large sample of clinical studies still need to be done before serum HSP27 could be used to conduct the diagnosis and treatment of lung cancer in clinical practice.

Yet, there are some deficiencies in this study. Firstly, because experimental specimens on tissue levels were mainly gotten from the surgical resection, this may lead to a selection bias of patients. Secondly, the number of study samples was relatively small, thus a larger number of samples should be required to get a more definitive conclusion. Thirdly, we did not study the molecular signal mechanism on the relationship between the HSP27 and NSCLC. Hence, mechanism research via RNA interference and HSP27 antibody neutralization need to be done for elucidating the role of HSP27 in NSCLC.

## Conclusions

We found that the expression of HSP27 was increased in lung cancer tissues and serum of NSCLC patients and that elevated HSP27 in NSCLC was associated with poorly differentiated cancer, lymphatic metastasis and advanced TNM stage. In addition, higher expression of HSP27 correlated with the shorter survival of patients with NSCLC. These results indicated that overexpression of HSP27 was potentially involved in the aggression and prognosis of lung cancer and that HSP27 may serve as a potential biomarker for diagnosis and prognosis of NSCLC.

## Methods and Materials

### Patient population

From January 2011 to December 2012, a total 76 confirmed patients with NSCLC were recruited in the study (Jining NO.1 People’s Hospital, Jining City, Shandong Province), including 38 patients with LSCC and 38 patients with LAC. We collected the matched fresh lung cancer tissues and normal adjacent tissues in surgery operation. The serum specimens of patients were gathered before receiving chemotherapy and surgery. Over the same period, the serum samples of 38 benign lung disease patients were gathered as control (59 ± 6.5 years) from the hospital mentioned above, including acute infectious diseases, chronic obstructive pulmonary disease, tuberculosis and interstitial diseases. Table 5 lists the clinical features of patients.

**Table 5:** Clinico-pathological features of included NSCLC patients. NSCLC, non-small cell lung cancer; Severe, >40 pack years; Moderate, 20.1–40 pack years; Slight, 0.1–20 pack years; LAC, lung adenocarcinoma; LSCC, lung squamous cell carcinoma.

**Items**	**Characteristics**	**Lung cancer (N = 76)**	**Non-lung cancer (N = 38)**
Sex			
	Male	60(78.95%)	20(52.6%)
	Female	16(21.05%)	18(47.4%)
Age			
	<60	34(44.7%)	23(60.5%)
	≥60	42(55.3%)	15(39.5%)
Pack years of smoking			
	>40	25(32.9%)	11(28.9%)
	20.1–40	2(2.6%)	3(7.9%)
	0.1–20	4(5.3%)	2(5.3%)
	0	45(59.2%)	22(57.9%)
Histology			
	LAC	38(50%)	
	LSCC	38(50%)	
Pathologic grade			
	Poorly differentiated	25(32.9%)	
	Moderately differentiated	22(28.9%)	
	Well-differentiated	29(38.2%)	
Clinical staging			
	IIA–IIIA	36(47.4%)	
	IIIB–IV	40(52.7%)	
Lymphatic invasion			
	Positive	50(65.8%)	
	Negative	26(34.2%)	

All samples were collected in accordance with the ethical guidelines, written informed consent was received. All patients were approached based on approved ethical guidelines, and those who agreed to participate in this study were required to sign consent forms. All the patients signed the informed consent before including the study. The study was approved by Research Ethics Committee of Jining NO.1 People’s Hospital (Jining City, Shandong Province). We confirmed that all methods were performed in accordance with the relevant guidelines and regulations.

### Pathological studies and tissue microarray (TMA) construction

Tissue microarray (TMA) blocks were prepared using lung cancer tissue and adjacent normal tissue from 76 lung cancer patients according to the method published previously^23^. The TMAs were constructed with a tissue array instrument (Beecher Instruments, Manual Tissue Arrayer, USA) by removal of a tissue core from the donor block using a thin-walled needle with an inner diameter of approximately 2.0 mm. Two core samples of each tumor were precisely placed into a recipient block at specifically assigned locations. Adjacent normal tissues were used as the control, which were also placed into the same recipient block. The array block was sectioned and leveled on the microscope slide and then baked in an oven and finally tested with routine H&E staining and IHC.

### Immunohistochemistry (IHC)

The expression of HSP27 in lung cancer tissues and adjacent normal tissues was tested by IHC method (Kit of Ultra-sensitive S-P; Fuzhou Maixin biotechnology company, China) according to a previous method that was described in published article^23^. The best dilution of anti-human HSP27 antibody is 1:100. The positive staining slice of pancreatic cancer served as a positive control; PBS was used instead of the primary antibody as the negative control. We evaluated the immunostaining of HSP27 according to a previous method described^2^. Cells that appeared to have brown-yellow granules in the nuclei or cytoplasm were considered to be positive cells. The staining was scored separately as follows: 0 for no staining in tumour cells; 1 for moderate staining; and 2 for intense staining of more than 75% of tumour cells; >1 was considered to be a positive result. Upon differences in scoring, the sections were evaluated in conjunction with the two pathologists.

### Real-time PCR

The vein blood of 2 mL was collected from each patient. The blood was centrifugated at 1,000 rpm at 4 °C for 5 min for three times. The serum was extracted and stored in ultra low temperature refrigerator at −80 °C. We used the silica gel column, ordinary trizol and strengthened silica gel column method to extract total RNA of serum, and evaluated the extraction efficiency. Then, we detected the target gene by reverse transcription and real-time PCR method. The conditions of amplification: pre-incubation at 94 °C for 6 min; 38 cycles at 95 °C for 5 s; 7 °C for 45 s; and 72 °C for 30 s. We analysed the quantity of mRNA using the 2^−ΔΔCt^ method. The GAPDH gene was served as the internal control. We designed the primer sequences of mRNA for detection molecular targets, housekeeper gene GAPDH: forward 5′-AACATCATCCCTGCCTCTACTG-3′; reverse 5′-CTCCGACGCCTGCTTCACC-3′; target gene HSP27: forward 5′-AAGGATGGCGTGGTGGAGATC-3′; reverse 5′-TCGTTGGACTGCGTGGCTAG-3′. We drew the dissolution curve by Bio-Rad iQ5, export dissolution curve and the Ct values (threshold value). Finally, we calculated the 2^−ΔΔCt^ values and analyzed the real time quantitative results.

### Statistical analysis

We used the Chi-square test, Fisher’s exact, and McNemar test to determine the association between the HSP27 expression and clinicopathological parameters of NSCLC. The expression level of serum HSP27 in different groups was compared using the Student’s *T*-test (matched samples), One-WAY ANOVA (single factor analysis), Kruskal Wallis Test (nonparametric analysis) and non-parametric rank correlation analysis. Survival curves derived from the Kaplan-Meier method were employed to disclose the risk of HSP27 expression to the survival of lung cancer patients. Cox proportional hazard regression model was utilized to determine the influence of each variable on the survival of lung cancer patients. We used the SPSS 17.0 software (SPSS, Chicago, IL, USA) to implement the statistical analyses. To assess the diagnostic potential of HSP27 in serum for lung cancer, we performed the receiver operator characteristic curve (ROC) analysis. All tests of statistical significance were two-sided, and a P-value < 0.05 was considered as statistically significant.
